# Response to correspondence on “Reproducibility of CRISPR-Cas9 methods for generation of conditional mouse alleles: a multi-center evaluation”

**DOI:** 10.1186/s13059-021-02320-3

**Published:** 2021-04-07

**Authors:** Channabasavaiah B. Gurumurthy, Aidan R. O’Brien, Rolen M. Quadros, John Adams, Pilar Alcaide, Shinya Ayabe, Johnathan Ballard, Surinder K. Batra, Marie-Claude Beauchamp, Kathleen A. Becker, Guillaume Bernas, David Brough, Francisco Carrillo-Salinas, Wesley Chan, Hanying Chen, Ruby Dawson, Victoria DeMambro, Jinke D’Hont, Katharine Dibb, James D. Eudy, Lin Gan, Jing Gao, Amy Gonzales, Anyonya Guntur, Huiping Guo, Donald W. Harms, Anne Harrington, Kathryn E. Hentges, Neil Humphreys, Shiho Imai, Hideshi Ishii, Mizuho Iwama, Eric Jonasch, Michelle Karolak, Bernard Keavney, Nay-Chi Khin, Masamitsu Konno, Yuko Kotani, Yayoi Kunihiro, Imayavaramban Lakshmanan, Catherine Larochelle, Catherine B. Lawrence, Lin Li, Volkhard Lindner, Xian-De Liu, Gloria Lopez-Castejon, Andrew Loudon, Jenna Lowe, Loydie Jerome-Majeweska, Taiji Matsusaka, Hiromi Miura, Yoshiki Miyasaka, Benjamin Morpurgo, Katherine Motyl, Yo-ichi Nabeshima, Koji Nakade, Toshiaki Nakashiba, Kenichi Nakashima, Yuichi Obata, Sanae Ogiwara, Mariette Ouellet, Leif Oxburgh, Sandra Piltz, Ilka Pinz, Moorthy P. Ponnusamy, David Ray, Ronald J. Redder, Clifford J. Rosen, Nikki Ross, Mark T. Ruhe, Larisa Ryzhova, Ane M. Salvador, Sabrina Shameen Alam, Radislav Sedlacek, Karan Sharma, Chad Smith, Katrien Staes, Lora Starrs, Fumihiro Sugiyama, Satoru Takahashi, Tomohiro Tanaka, Andrew Trafford, Yoshihiro Uno, Leen Vanhoutte, Frederique Vanrockeghem, Brandon J. Willis, Christian S. Wright, Yuko Yamauchi, Xin Yi, Kazuto Yoshimi, Xuesong Zhang, Yu Zhang, Masato Ohtsuka, Satyabrata Das, Daniel J. Garry, Tino Hochepied, Paul Thomas, Jan Parker-Thornburg, Antony D. Adamson, Atsushi Yoshiki, Jean-Francois Schmouth, Andrei Golovko, William R. Thompson, K. C. Kent Lloyd, Joshua A. Wood, Mitra Cowan, Tomoji Mashimo, Seiya Mizuno, Hao Zhu, Petr Kasparek, Lucy Liaw, Joseph M. Miano, Gaetan Burgio

**Affiliations:** 1 Mouse Genome Engineering Core Facility, Vice Chancellor for Research Office, University of Nebraska Medical Center, Omaha, NE USA; 2 Developmental Neuroscience, Munroe Meyer Institute for Genetics and Rehabilitation, University of Nebraska Medical Center, Omaha, NE USA; 3 Transformational Bioinformatics, Health and Biosecurity Business Unit, CSIRO, Sydney, Australia; 4 Department of Immunology and Infectious Disease, the John Curtin School of Medical Research, The Australian National University, Canberra, Australia; 5 Texas A&M Institute for Genomic Medicine (TIGM), Texas A&M University, College Station, TX 77843 USA; 6 Department of Immunology, Tufts University School of Medicine, Boston, USA; 7 RIKEN BioResource Research Center, Tsukuba, Ibaraki 305-0074 Japan; 8 Department of Biochemistry and Molecular Biology, University of Nebraska Medical Center, Omaha, NE USA; 9 Departments of Anatomy and Cell Biology, Human Genetics and Pediatrics, Research Institute McGill University Health Center (RI-MUHC), Montreal, Canada; 10 Maine Medical Center Research Institute (MMCRI), Scarborough, ME USA; 11 Transgenesis and Animal Modeling Core Facility, Centre de Recherche du Centre Hospitalier Universitaire de Montreal (CRCHUM), Montreal, Canada; 12 Division of Neuroscience and Experimental Psychology, School of Biological Sciences, Faculty of Biology, Medicine and Health, Manchester Academic Health Science Centre, University of Manchester, AV Hill Building, Oxford Road, Manchester, M13 9PT UK; 13 School of Medicine, Indiana University, Indianapolis, IN 46202 USA; 14 South Australian Health & Medical Research Institute and Department of Medicine, University of Adelaide, Adelaide, Australia; 15 Transgenic mouse core facility, VIB Center for Inflammation Research, Ghent, Belgium; 16 Department of Biomedical Molecular Biology, Ghent University, Ghent, Belgium; 17 Unit of Cardiac Physiology, School of Medical Sciences, Manchester Academic Health Science Center, University of Manchester, Manchester, UK; 18 High-Throughput DNA Sequencing and Genotyping Core Facility, Vice Chancellor for Research Office, University of Nebraska Medical Center, Omaha, USA; 19 University of Rochester Medical Center, Rochester, NY 14642 USA; 20 Division of Evolution and Genomic Sciences, School of Biological Sciences, Faculty of Biology, Medicine and Health, Manchester Academic Health Science Centre, University of Manchester, Manchester, UK; 21 Transgenic Unit core facility, Faculty of Biology, Medicine and Health, University of Manchester, Manchester, UK; 22 Department of Basic Medicine, Division of Basic Medical Science and Molecular Medicine, School of Medicine, Tokai University, 143, Shimokasuya, Isehara, Kanagawa 259-1193 Japan; 23 Department of Medical Data Science, Osaka University Graduate School of Medicine, Suita, Japan; 24 The University of Texas, MD Anderson Cancer Center, Houston, TX USA; 25 Division of Cardiovascular Sciences, School of Medical Sciences, Faculty of Biology, Medicine and Health, The University of Manchester AND Manchester Heart Centre, Manchester University NHS Foundation Trust, Manchester Academic Health Science Centre, Manchester, UK; 26 Department of Frontier Science for Cancer and Chemotherapy, Osaka University Graduate School of Medicine, Osaka, Japan; 27 The Institute of Experimental Animal Sciences, Osaka University Graduate School of Medicine, Osaka, Japan; 28 Centre de Recherche du Centre Hospitalier Universitaire de Montreal (CRCHUM), Montreal, Canada; 29 Children’s Research Institute Mouse Genome Engineering Core, University of Texas Southwestern Medical Center, Dallas, TX 75390 USA; 30 Manchester Collaborative Centre for Inflammation Research (MCCIR), School of Biological Sciences, Faculty of Biology, Medicine and Health, The University of Manchester, Manchester, UK; 31 Centre for Biological Timing, School of Medical Sciences, Faculty of Biology, Medicine and Health, University of Manchester, Manchester, UK; 32 Center for Matrix Biology and Medicine, Graduate School of Medicine, Tokai University, Isehara, Kanagawa 259-1193 Japan; 33 Department of Molecular Life Science, Division of Basic Medical Science and Molecular Medicine, School of Medicine, Tokai University, 143, Shimokasuya, Isehara, Kanagawa 259-1193 Japan; 34 Laboratory of Molecular Life Science, Foundation for Biomedical Research and Innovation, Kobe, Japan; 35 Department of Laboratory Animal Science, Support Center for Medical Research and Education, Tokai University, 143, Shimokasuya, Isehara, Kanagawa 259-1193 Japan; 36 Oxford Centre for Diabetes, Endocrinology and Metabolism, University of Oxford, Oxford, OX37LE UK; 37 Mouse Biology Program, University of California, Davis, USA; 38 Laboratory of Transgenic Models of Diseases and Czech Centre for Phenogenomics, Institute of Molecular Genetics of the Czech Academy of Sciences, Prague, Czech Republic; 39 College of Osteopathic Medicine, Marian University, Indianapolis, IN 46222 USA; 40 Laboratory Animal Resource Center, University of Tsukuba, Tsukuba, Japan; 41 Department of Gastroenterology and Metabolism, Nagoya City University Graduate School of Medical Sciences, Nagoya, Japan; 42 Department of Physical Therapy, School of Health and Human Sciences, Indiana University, Indianapolis, IN 46202 USA; 43 Lillehei Heart Institute Regenerative Medicine and Sciences Program, University of Minnesota, Minneapolis, MN USA; 44 Paul and Sheila Wellstone Muscular Dystrophy Center, University of Minnesota, Minneapolis, MN USA; 45 Department of Surgery, School of Medicine, University of California, Davis, Davis, USA; 46 McGill Integrated Core for Animal Modeling (MICAM), Montreal, Canada

We would like to thank the Yang et al. (2013) authors for their comments and debate on optimal methods for mouse transgenesis. The two Jaenisch laboratory studies published in *Cell* in 2013 were ground-breaking, demonstrating for the first time proof of principle CRISPR mediated gene editing in the mouse zygote to generate knockout and conditional alleles, and caused much excitement in the transgenic mouse community.

However, over several years and in many laboratories, the reality did not match the excitement when it came to generating conditional alleles in a single step. While it is true that the 2-guides 2-oligo approach can work in certain circumstances (members of our own consortium reported some success with this method), the efficiencies reported in Yang et al. (2013) do not bear out across multiple gene targets. Indeed, as the comments from Yang et al. point out, they themselves have performed further reproducibility experiments on the *Mecp2* locus (their point #1) and these unpublished results fail to reproduce the 16% efficiency from their original publication. And our study is not the first time concerns have been raised as to the efficiency of the 2-guides 2-oligo method, with anecdotal reports from others in the transgenic community (Science; 2016. doi:10.1126/science.aal0334 [doi.org]), which stated that *“What was disappointing is none of us could reproduce at the efficiencies reported by Jaenisch. … It works at 1% or 2% at JAX and a lot of projects are failing. It’s really not proven to be a robust method.” *And the Yang et al. group’s response was that *“The paper reported what we found,” Jaenisch says. “Now, we see there are issues”.*

In regard to their point #2, regrettably, details of concentrations of reagents used were not reported in Yang et al. (2013). The authors, in this correspondence, now state that they had provided concentrations in their other report [ref #4], but this reference (#4) does not describe generation of conditional alleles, and therefore, the experimental conditions of this paper (ref #4) cannot be extrapolated for generating the conditional alleles. Also, because such critical details were unavailable in the Yang et al. paper (ref #1), some of us had contacted the authors asking for tips on how to get their method to work, but we received no response. This oversight of the authors (in failing to describe the concentrations in the original paper) indeed allowed us to assess efficiencies using a range of conditions on many loci, both lower and greater than the now revealed conditions from Yang et al. (2013), and using different delivery platforms (microinjection, electroporation), the results of which further confirm that the Yang et al. method is not efficient as it was originally reported. We suggest the reader to refer to the extensive data in our additional file 1 (supplementary data file) where we show that the wide range of reagent concentrations does not affect the efficiency of the Yang et al. method. Furthermore, Hatada’s group (Horii et al.) attempted to reproduce the *Mecp2* experiments, and they reported either very low efficiency or very high toxicity when the concentration of reagents was in the higher range. See Table 1 in Horii et al.; the concentration of 50/12/100 produced only 2% efficiency whereas the concentration 100/24/200 led to the death of nearly 90% of embryos, and the authors were unable to determine the method’s efficiency at this higher concentration.

In regard to their point #3, the authors speculate that Piezo-driven zygote injection may contribute to the difference of success rates. It would be necessary to examine this speculation by comparing the efficiencies of Piezo-driven and pronuclear injection methods side-by-side for a few loci. Because efficiencies at different genomic loci often vary highly (which the Yang et al. authors state in their paragraph below point #3), it would be ideal to gather such side-by-side data for at least 6 to 10 loci or more to ensure reproducibility. Otherwise, the assumption remains speculative.

Further, the authors in the paragraph below their point #3 suggest that their original method may not be efficient on other loci by stating that *“it would be premature for scientific community to assume that their method would work on other loci” *indicating that their study was too underpowered for routine use in core facilities. We discussed this specific point (underpower) in our paper: we suggest the reader to refer to the discussion section of our paper from the sentence that reads *‘While many published methods are reproducible (as evidenced by their wide usage), the research community often encounters issues in reproducing some published methods.’*

Lastly, our observations call into question the robustness of the approach and its suitability for widespread use. Additionally, we evaluated alternative methods in parallel to report improved efficiencies across several gene targets using one-donor methods. It is vital we hold published methodologies to the highest possible standards, especially in the field of mouse transgenesis, where widespread adoption of low efficiency genetic manipulation strategies can have ethical consequences on the number of animals used in research. Science in general currently has a reproducibility crisis (https://www.nature.com/collections/prbfkwmwvz [nature.com]), and it is our responsibility as scientists that published methods are robustly tested and that the results from higher-powered analyses, which can at times be contradictory, are themselves published.

